# Response mechanism of hepatorenal metabolic barrier function to drugs and environmental poisons and intervention strategy of natural products

**DOI:** 10.3389/fphar.2026.1763782

**Published:** 2026-05-04

**Authors:** Zhen Li, Yi Li, Junfeng Zhu, Zhiqiang Zheng, Wenqiang Xie, Che Xu

**Affiliations:** Yueyang Hospital of Integrated Traditional Chinese and Western Medicine, Shanghai University of Traditional Chinese Medicine, Shanghai, China

**Keywords:** drug toxicants, environmental toxicants, intervention mechanism, hepatic metabolic barrier, renal metabolic barrier, natural products

## Abstract

Liver and kidney, as the core metabolic detoxification organs of human body, rely on specific biofilm barrier and metabolic enzyme system to form metabolic barrier respectively. This metabolic barrier is the key defense line to resist the invasion of drugs and environmental poisons. There are significant differences in the structural function and toxic response mechanism of the metabolic barrier between the liver and kidney. This review explains the core composition and physiological function of the hepatorenal metabolic barriers respectively. It also systematically analyzes the specific mechanisms of toxic damage to these barriers. The core of liver injury is the destruction of hepatic sinus barrier, disorder of metabolic enzyme expression and bile excretion disorder. Renal injury focuses on glomerular filtration barrier injury, abnormal renal tubular transport function and renal interstitial inflammatory reaction. In this review, the specific protective value and intervention mechanism of natural products such as herbal components, single Chinese medicinal herbs and Chinese medicinal prescriptions on hepatorenal metabolic barrier damage were summarized. This provides targeted theoretical support for the development of hepatorenal protective drugs.

## 1 Introduction

**FIGURE 1:**
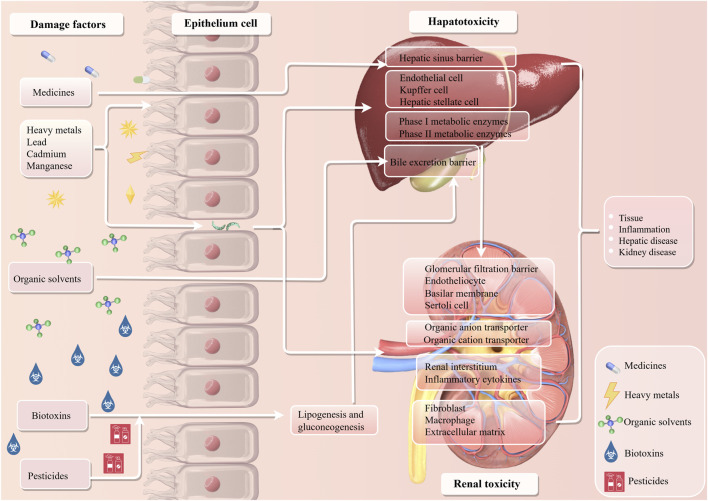
Mechanism of drug or environment-induced hepatorenal toxicity.

In modern society, drugs are widely used in the clinical treatment of diseases, and environmental pollution caused by industrial development has intensified. This exposes people to a variety of environmental poisons on a continuous basis. Liver and kidney are the core organs of human metabolism and detoxification. They can maintain the homeostasis of the internal environment and resist the invasion of exogenous substances through their own metabolic barriers. There are significant differences in their structure, physiological functions and poison response mechanisms (Luo et al., 2022; Chrysopoulou and Rinschen, 2024). The hepatic metabolic barrier is centered on the hepatic sinus, liver cell metabolic enzyme system and bile excretion system. The kidney metabolic barrier, by contrast, depends on the glomerular filtration barrier (GFB), renal tubular transport system and renal interstitial vascular network (He Q. et al., 2024; Faivre et al., 2024). After drugs and environmental poisons enter the body, they can interfere with the enzyme activity and transport function of hepatorenal metabolic barriers and cause organ-specific damage (Zuo et al., 2024). This regulatory effect may further induce hepatorenal injury, as illustrated in Figure 1.

In recent years, the molecular mechanism of hepatorenal metabolic barrier damage has been gradually deepened. However, due to the significant difference between the two damage laws, mixed discussion tends to cover up their specific characteristics and affect the clarity of research logic. Because of the unique advantages of wide sources and multi-target regulation, natural products show targeted protection potential in the intervention of hepatorenal metabolic barrier injury. They can exert this effect by regulating oxidative stress (OS) and repairing the barrier structure respectively (Sha et al., 2021; Wang D. et al., 2024).

Therefore, this review combs the structure and function of hepatorenal metabolic barriers and the molecular mechanisms of toxicity-induced damage. It also systematically summarizes the specific intervention strategies of natural products for such injuries. This review aims to provide theoretical and practical basis for the accurate prevention and control of hepatorenal damage induced by drugs and environmental poisons.

## 2 Structure and function of hepatic metabolic barrier

**FIGURE 2:**
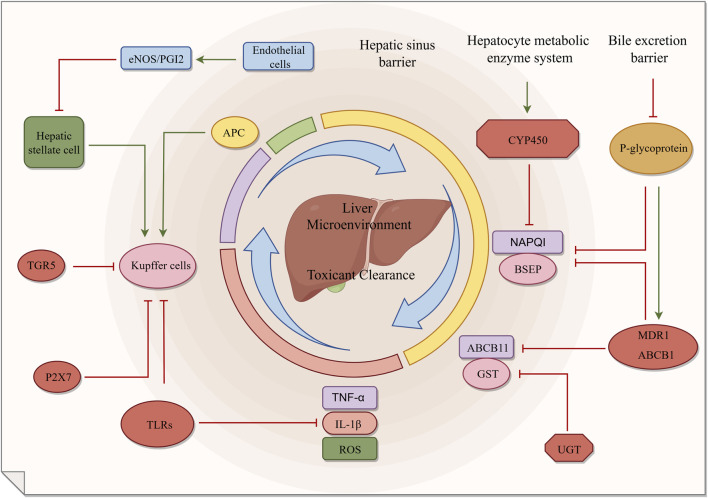
Structure and function of hepatic metabolic barrier.

Hepatic metabolic barrier is an important defense line for human body to resist exogenous poisons. It consists of hepatic sinus barrier, liver cell metabolic enzyme system and bile excretion barrier (Gracia-Sancho et al., 2021; Varanasi et al., 2025; Wang G. et al., 2023). These three layers work together to realize the identification, biotransformation and excretion of poisons. The functional integrity of the barrier directly affects the efficacy and toxicity risk of drugs, and is closely related to the pathogenesis and progress of various liver diseases (Table 1; Figure 2) (Gil-Pitarch et al., 2024).

**TABLE 1:** Comparison of structure and function of hepatorenal metabolic barriers.

Barrier type	Core components	Main functions	Key characteristics
Hepatic Metabolic Barrier	Hepatic sinus barrier (endothelial cells, Kupffer cells, hepatic stellate cells), hepatocyte metabolic enzyme system (CYP450, UGT, etc.), bile excretion barrier (transporters such as MDR1, MRP2)	Toxicant recognition, biotransformation, bile excretion, and resistance to exogenous substance invasion	Multi-layered structural synergy for detoxification, with final toxicant clearance dependent on bile excretion
Renal Metabolic Barrier	Glomerular filtration barrier (endothelial cells, basement membrane, podocytes), renal tubular system (OATs, OCTs transporters), renal interstitial vascular barrier	Toxicant filtration, reabsorption regulation, directional excretion, and maintenance of internal environmental homeostasis	Filtration-transport as the core, with barrier function maintained by hemodynamics

The hepatic sinus and its surrounding structures form the primary defense line against toxicants in the liver, with liver sinusoidal endothelial cells, Kupffer cells (KCs) and hepatic stellate cells (HSCs) exerting a synergistic defensive effect (Wang K. et al., 2024; Li W. et al., 2023; Yan M. et al., 2025). Liver sinusoidal endothelial cells are porous with fenestrae measuring approximately 100–150 nm in diameter. These fenestrae allow the free passage of plasma components while restricting the extravasation of blood cells. This structural feature enables the cells to balance the efficiency of material exchange with the selectivity of the barrier (Wu Q. et al., 2024). KCs are the resident macrophages of the liver, accounting for 80%–90% of the total number of fixed macrophages in the whole body (Li et al., 2022a). It recognizes pathogens and poisons through Toll-like receptors (TLRs) on its surface and initiates phagocytosis and immune responses. It can thus timely remove poisons present in the circulation (Xu L. et al., 2021; Jaeschke and Ramachandran, 2024). HSCs store vitamin A under normal physiological conditions. Upon liver injury, these cells become activated and transform into myofibroblasts, thereby participating in the progression of hepatic fibrosis (Kamm and McCommis, 2022). These three cells maintain the stability of the liver microenvironment through intercellular communication.

Hepatocyte metabolic enzyme system is the core of liver detoxification function. Phase I metabolic enzymes are represented by the cytochrome P450 (CYP450) superfamily. Subtypes such as cytochrome P450 family 3 subfamily A member 4 (CYP3A4) and cytochrome P450 family 2 subfamily E member 1 (CYP2E1) are involved in the oxidative metabolism of approximately 75% of clinical drugs (Liu R. et al., 2023). Some metabolic processes will produce highly active intermediates, and N-acetyl-p-benzoquinone imine (NAPQI) produced by metabolism of acetaminophen (APAP) by CYP2E1 is a typical example (Fang et al., 2023). When NAPQI exceeds the detoxification ability of glutathione (GSH), it will induce acute liver injury (Sun et al., 2022). Phase II metabolic enzymes reduce the lipid solubility of toxicants through binding reaction (Neves-Silva et al., 2025). Uridine diphosphate glucuronosyltransferase (UGT) and glutathione S- transferase (GST) can significantly improve the water solubility and excretion efficiency of poisons (Alarcan et al., 2017).

Bile excretion barrier is the key way for the liver to excrete poisons. Hepatocytes will excrete metabolized poisons into bile with the help of various transporters (Horiuchi et al., 2022). These transporters include P-glycoprotein, multidrug resistance protein 1 (MDR1/ABCB1), multidrug resistance-related protein 2 (MRP2/ABCC2), bile salt export pump (BSEP/ABCB11) and so on (Dietrich et al., 2003; Norman, 2020). MRP2 is responsible for mediating the excretion of organic anions and drug metabolites, while BSEP is responsible for the excretion of bile acids (Wang P. et al., 2025; Wang M. Q. et al., 2024). Abnormal transporter function can lead to cholestasis, for example, mutation of MRP2 gene can lead to Dobbin-Johnson syndrome (Junge et al., 2021). Some poisons can be reabsorbed into the enterohepatic circulation after being excreted in bile. This reabsorption prolongs their *in vivo* exposure time and impairs the body’s detoxification efficiency (Remetic et al., 2022).

The three layers of hepatic metabolic barrier work together to construct a complete liver detoxification system. However, genetic polymorphism, drug interaction and chronic injury may destroy the integrity of this barrier. And then induce drug-induced liver injury (DILI), hepatic fibrosis and other diseases. Therefore, it is of great significance to deeply analyze its injury mechanism for the prevention and treatment of liver diseases.

## 3 Structure and function of renal metabolic barrier

**FIGURE 3:**
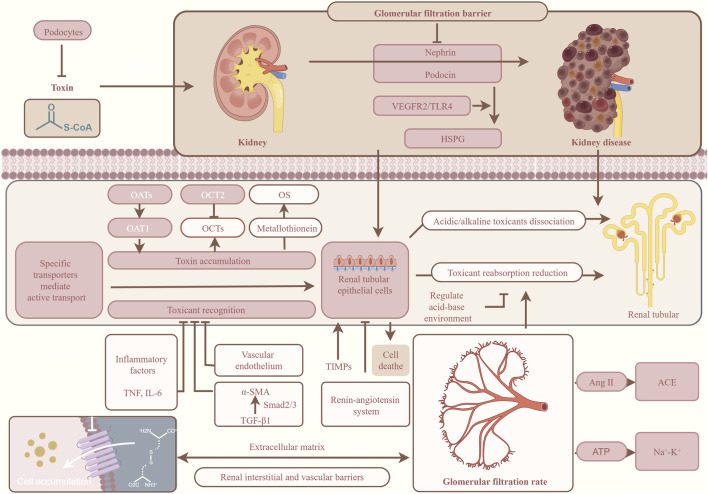
Structure and function of renal metabolic barrier.

Renal metabolic barrier is the core defense line for the body to excrete waste and maintain the homeostasis of the internal environment. It relies on GFB, renal tubular system and renal interstitial vascular barrier to complete the filtration, reabsorption regulation and directional excretion of poisons (Li X. et al., 2023; Wang X. L. et al., 2024; Jiang et al., 2024). The integrity of this barrier directly determines the clearance efficiency of renal toxicants, and is closely related to the pathogenesis and progress of acute and chronic nephropathy (Table 1; Figure 3) (Lash, 2021; Foresto-Neto et al., 2024).

GFB is the first barrier of kidney to resist poison, which consists of three layers: endothelial cells, basement membrane and podocytes (Daehn and Duffield, 2021; Li M. et al., 2024). The surface aperture of glomerular endothelial cells is 50–100 nm in diameter, which can allow small molecules to pass through and block blood cells and macromolecular proteins at the same time (Jia L. et al., 2025). The middle basement membrane forms a double barrier of charge and pore size, negatively charged heparan sulfate can repel albumin, and collagen network can limit macromolecular filtration (Naylor et al., 2021). Podocytes form a hole membrane through the staggered foot processes, which is composed of proteins such as nephrin and podocin (Seifert et al., 2023; Liang et al., 2024). The hole film can precisely control the passage of substances and prevent the deposition of harmful substances (Agarwal et al., 2021). Any damage of the three-layer structure can lead to proteinuria and renal function damage.

Renal tubular system is the core place for kidney to metabolize and excrete poisons. Proximal renal tubular epithelial cells highly express organic anion transporters (OATs) and organic cation transporters (OCTs). These transporters secrete endogenous metabolites and exogenous poisons along a reversible concentration gradient (Granados and Nigam, 2024; Han et al., 2021). Among them, organic anion transporter 1 (OAT1) and organic anion transporter 3 (OAT3) are responsible for recognizing organic acids such as methotrexate and nonsteroidal anti-inflammatory drugs, while organic cation transporter 2 (OCT2) mediates the excretion of organic bases such as metformin and cimetidine (Wang C. et al., 2023; Parvez et al., 2017; Sandoval et al., 2019; Wu H. et al., 2025). However, the strong reabsorption function of proximal renal tubules easily leads to the accumulation of poisons in the body. Heavy metals such as lead and cadmium can combine with metallothionein, accumulate in renal tubular cells and induce OS, which eventually leads to renal tubular necrosis (Zavala-Guevara et al., 2021; Ikebuchi et al., 1986). Distal renal tubules and collecting tubes affect the pH environment of toxicant excretion by regulating ion transport, and promote the dissociation and excretion of acidic or alkaline toxicants (Tabibzadeh and Crambert, 2023; Wang S. et al., 2024).

Renal interstitial and vascular barriers maintain renal hemodynamics and electrolyte balance. Renal interstitium provides structural support for renal tubules, and participates in the inflammatory reaction and fibrosis process of kidney (Yan et al., 2021; Lu et al., 2018). In sepsis, inflammatory factors will destroy the tight junction of vascular endothelium. This will increase capillary permeability, and then induce acute kidney injury (AKI) (Chen et al., 2025). Renal interstitial vascular network maintains the stable perfusion pressure of glomerulus through renin-angiotensin system (RAS). If there is hemodynamic disorder, the glomerular filtration rate (GFR) will be reduced, which will affect the excretion of poisons (Rahaman et al., 2021).

## 4 Damage mechanism of drugs and environmental poisons to hepatic metabolic barrier

Drug abuse and exposure to environmental poisons are easy to attack the hepatic metabolic barrier. Its damage mechanism is diverse, including OS, starting inflammatory reaction, interfering with metabolic enzyme function and destroying barrier structure (Table 2). These mechanisms will lead to the decline of liver detoxification function, and then lead to specific liver injury (Aljobaily et al., 2020; Zhao et al., 2024; Wang X. et al., 2025). Clarifying the damage law of toxicants to hepatic metabolic barrier can provide targeted basis for the prevention and treatment of liver diseases.

**TABLE 2:** Comparison of damage mechanism of drugs and environmental toxicants on hepatorenal metabolic barriers.

Injury dimension	Injury mechanism of hepatic metabolic barrier	Injury mechanism of renal metabolic barrier	Typical toxicants/Drugs
Oxidative stress and Mitochondrial Damage	The activation of NADPH oxidase leads to the excessive production of ROS, and the inhibition of SOD and GSH-Px activities hinders the elimination of ROS. Excessive ROS damages the inner membrane of mitochondria, which leads to lipid peroxidation, membrane potential disintegration, mPTP opening, and the release of apoptosis-promoting factors such as cytochrome C, which initiates hepatocyte injury and apoptosis	After entering renal tubular epithelial cells through OATs, mitochondrial damage and insufficient ATP production lead to reabsorption disorder. Excessive ROS also activates inflammatory pathways to form a vicious circle of oxidative stress-inflammation, which aggravates the damage of renal metabolic barrier and leads to acute renal injury	Acetaminophen, cisplatin, heavy metals
Inflammation Reaction and Hepatic Sinus Barrier Permeability	Activating TLR4 on KCs surface, releasing pro-inflammatory factors through MyD88 pathway to induce hepatocyte apoptosis, and long-term inflammation activates HSCs to synthesize a large amount of extracellular matrix to cause liver fibrosis, destroy hepatic sinus barrier, aggravate hepatocyte hypoxia, and finally lead to liver failure	Secreting chemokines such as MCP-1 and RANTES, recruiting macrophages to infiltrate renal interstitium, releasing protease and ROS to damage renal tubules, at the same time up-regulating ICAM-1 to enhance immune adhesion, accelerating renal fibrosis through inflammatory cascade reaction, and improving the permeability of renal metabolic barrier	Gentamicin, heavy metals
Abnormal Transport/Metabolic Function	Inhibiting the activities of CYP3A4 and GST, interfering with CYP2E1-mediated metabolic pathway, disrupting the balance of liver detoxification, and also affecting the expression of metabolic enzyme genes, resulting in toxic accumulation, production of toxic intermediates such as NAPQI, and finally causing liver injury and even liver failure	Inhibit the activity of OATs and OCTs transporters, combine with sulfhydryl groups of nephrin and podocin proteins and change their spatial conformation, resulting in podocyte shedding and impaired glomerular filtration barrier structure and function	Heavy metals, chloramphenicol, acetaminophen
Structural Damage	Embedding mRNA interferes with the proliferation and repair of hepatic sinusoidal endothelial cells, and produces ROS to attack cytoskeleton proteins, causing structural damage, plasma protein leakage, complement activation and inflammation	Transporters bind to nephrin and podocin sulfhydryl groups in podocytes, resulting in abnormal conformation, foot process shedding, destruction of filtration membrane and proteinuria	5-flurouracil, cisplatin, gentamicin

Abbreviations: GSH-Px, glutathione peroxidase; SOD, superoxide dismutase; ROS, reactive oxygen species; GST, glutathione S- transferase.

### 4.1 Oxidative stress and mitochondrial damage

Oxidative stress and subsequent mitochondrial damage are the key pathological initiating factors of toxicity-induced hepatic metabolic barrier damage. These changes lead to cascading hepatocellular damage and ultimately impair hepatic barrier function. OS is the core initial link of toxic damage to hepatic metabolic barrier. Poisons generate excessive reactive oxygen species (ROS) by activating nicotinamide adenine dinucleotide phosphate (NADPH) oxidase and simultaneously inhibit the activities of superoxide dismutase (SOD) and glutathione peroxidase (GSH-Px). This dual effect blocks the normal clearance of ROS in the body (Liu F. C. et al., 2023). Excessive ROS will attack the inner membrane of mitochondria and trigger lipid peroxidation, resulting in the collapse of mitochondrial membrane potential and the opening of permeability transition pore (mPTP) (Liu Z. et al., 2024; Shen et al., 2024). This process will release apoptosis-promoting factors such as cytochrome C, and finally start the process of hepatocyte injury (Zhu L. et al., 2023). Heavy metals can induce OS, destroy antioxidant defense system, interfere with cell signal pathway and mitochondrial function, and cause liver cell injury and apoptosis. Long-term exposure to heavy metals will also aggravate hepatic fibrosis, and then induce pathological damage to the liver (Jomova et al., 2025).

### 4.2 Inflammation and increased permeability of hepatic sinus barrier

Persistent inflammation-driven hepatic sinusoidal barrier dysfunction represents a pivotal pathological hallmark of drug- and environmental toxicant-induced liver injury. Activation of KCs triggers a cascade of inflammatory responses that disrupt hepatic sinus barrier function, drive hepatic fibrosis, and ultimately lead to severe hepatic failure. KCs are the starting core of liver inflammatory reaction. Stimulated by gentamicin and other poisons, TLR4 on KCs surface is activated, and TNF-α, IL-6 and other pro-inflammatory factors are secreted through MyD88-dependent pathway (Yang et al., 2022; Fujiwara et al., 2012). These pro-inflammatory factors can not only directly induce hepatocyte apoptosis, but also upregulate the expression of Fas/FasL, and further promote the programmed death of liver cells (Chen et al., 2023). Long-term inflammatory stimulation will activate HSCs and make them synthesize extracellular matrix in large quantities (Xu et al., 2024; Abd El-et al., 2022). These matrices will form fibrous septa and gradually develop into cirrhosis, which will hinder the material exchange of hepatic sinus (Zhu X. et al., 2023). Finally, the hypoxia state of liver cells is aggravated, leading to liver failure.

### 4.3 Metabolic enzyme dysfunction and poison accumulation

Poisons destroy the balance of liver detoxification by inhibiting enzyme activity or interfering with metabolic pathways. Chloramphenicol can bind to the active site of CYP3A4, inhibit its catalytic function, and then prolong drug half-life, leading to toxic accumulation (Park et al., 2003). Cadmium ions can form covalent bonds with the sulfhydryl groups in the active center of GST, directly blocking the detoxification process of phase II metabolism (Jomova et al., 2025; Espinoza et al., 2012). When APAP is excessive, the glucuronidation and sulfation pathways will reach saturation. A large number of unmetabolized APAP is metabolized by CYP2E1, resulting in excessive toxic intermediate NAPQI (Torres et al., 2019). NAPQI will deplete GSH in the body, and then covalently bind with hepatocyte macromolecules, and finally induce acute liver failure (Jaeschke et al., 2021). In addition, poisons can interfere with the expression of metabolic enzyme genes, leading to the decline of curative effect or the formation of new toxic products (Yan H. et al., 2025).

### 4.4 Specific damage of liver barrier structure

The structural damage of hepatic sinus is a typical feature of toxic hepatic metabolic barrier damage. This damage destroys the structural integrity of the liver barrier and triggers a series of inflammatory and fibrotic reactions. These reactions further aggravate liver injury. Chemotherapeutic drugs such as 5-flurouracil can specifically destroy hepatic sinus endothelial cells. Its damage mechanism is mainly reflected in two aspects. On the one hand, it interferes with the process of cell proliferation and repair by embedding mRNA (Pilgrim et al., 2011). On the other hand, ROS will be generated to attack the skeleton protein, which will lead to the morphological changes of endothelial cells and cracks in the cell layer (El-Sayyad et al., 2009; Cao et al., 2023). This structural damage will lead to plasma protein leakage, which will activate the complement system and further aggravate the inflammatory reaction (El-Sayyad et al., 2009; Fernández et al., 2019). Long-term endothelial cell injury can induce capillarization of hepatic sinuses and accelerate the process of hepatic fibrosis (He S. et al., 2024).

## 5 Damage mechanism of drugs and environmental poisons to renal metabolic barrier

Drugs and environmental poisons exert distinct specific damage on the renal metabolic barrier. Their damage mainly targets the GFB, renal tubules and renal interstitium, and impairs renal function through multiple mechanisms including OS, inflammatory reactions and abnormal transporter function (Ricciardi and Gnudi, 2021; Zeisberg and Kalluri, 2015; Liu et al., 2018; Wu Q. et al., 2025). Compared with the liver, there are significant differences in the target and pathological process of renal injury (Table 2).

### 5.1 Oxidative stress and mitochondrial dysfunction

Oxidative stress (OS) and mitochondrial dysfunction are critical pathological mediators underlying renal metabolic barrier injury caused by chemotherapeutic drugs and toxic substances. Chemotherapeutic drugs such as cisplatin (CDDP) can enter renal tubular epithelial cells through OATs (Veiga-Matos et al., 2020). Once it enters the cell, it specifically induces mitochondrial damage and leads to insufficient ATP production. This in turn inactivates energy-dependent transporters such as Na+/K+-ATPase (Lin et al., 2023; Xin et al., 2025). This process will lead to renal tubular reabsorption dysfunction, and eventually progress to AKI (Yu et al., 2023). At the same time, excessive ROS can also activate the inflammatory signaling pathway, forming a vicious circle of “oxidative stress-inflammation.” This circulation will further aggravate renal injury (Ming et al., 2022; Jin et al., 2023).

### 5.2 Inflammatory reaction and renal barrier permeability

Inflammatory responses are a central pathological link in the elevation of renal metabolic barrier permeability. These responses disrupt the structural and functional integrity of the renal barrier and drive the progression of various renal injuries such as focal and segmental glomerulosclerosis (FSGS) and interstitial nephritis. In FSGS, CDDP induces OS in glomerular podocytes, leading to decreased expression of nephrin and podocin (Hye Khan et al., 2025; Abdel-Aal et al., 2025). This impairs the function of the filtration barrier, causes proteinuria and activates the complement system (Liu J. et al., 2022; Gong et al., 2023). Drug hypersensitivity can induce renal tubular epithelial cells to secrete chemokines such as monocyte chemoattractant protein-1 (MCP-1) and regulated on activation, normal T cell expressed and secreted (RANTES) (Tang et al., 2020). These chemokines will recruit macrophages to infiltrate renal interstitium, and then induce acute interstitial nephritis (AIN) (Tsai et al., 2025). Infiltrated immune cells will release protease and ROS, which will directly damage renal tubules (Liu Y. et al., 2017). At the same time, it can also upregulate intercellular adhesion molecule 1 (ICAM-1) expression, enhance immune cell adhesion, and finally accelerate the process of renal fibrosis (Niu et al., 2025).

### 5.3 Transporter dysfunction and toxin accumulation

Renal tubular transporter dyfunction is the key to the accumulation of renal toxins. Heavy metals, chemotherapy drugs and other substances can inhibit the activities of OATs and OCTs, thus hindering the secretion and excretion of poisons. They can also enter renal tubular cells via transporters and accumulate continuously in the cells (Veiga-Matos et al., 2020). Heavy metals such as mercury can selectively attack glomerular podocytes and combine with sulfhydryl groups of nephrin and podocin. This combination will change the spatial conformation of the two proteins and induce podocyte shedding (Jomova et al., 2025). In turn, it leads to the loss of GFB function and a large number of proteinuria, which is a typical feature of nephrotic syndrome (NS) (Raglianti et al., 2024). Factors released after podocyte injury can recruit inflammatory cells to further destroy glomerular structure (Liu X. Q. et al., 2022). Long-term proteinuria will overload renal tubular protein reabsorption and accelerate renal fibrosis and renal failure (Bailey et al., 2022).

### 5.4 Specific damage of renal barrier structure

The specific damage of renal barrier structure focuses on GFB, renal tubular epithelial cells and renal interstitial blood vessels, showing obvious toxicity and pathological specificit. Mercury, cadmium and other heavy metals enter the body via transporters. They bind to the sulfhydryl groups of nephrin and podocin proteins in podocytes, leading to abnormal protein conformation and foot process detachment. This disrupts the integrity of the filtration membrane, induces proteinuria and forms a vicious circle (Zhang K. et al., 2024; Wang and Mao, 2016). CDDP, gentamicin and other agents damage the renal tubular cytoskeleton via OATs, with prolonged stimulation able to induce renal interstitial fibrosis. Inflammatory factors in sepsis can also disrupt the vascular connections within the renal interstitium. This pattern of injury differs significantly from that of hepatic sinusoidal capillarization (Susa et al., 2023; Flannery et al., 2025).

## 6 Intervention strategies and mechanisms of natural products for hepatorenal metabolic barrier injury

**FIGURE 4:**
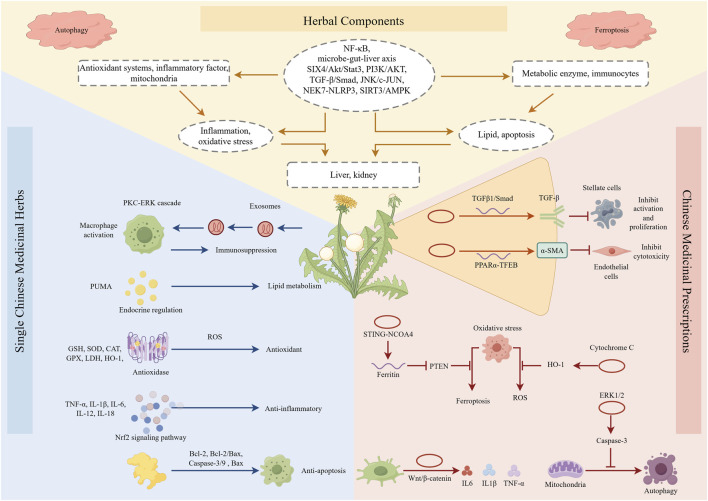
Intervention strategies and mechanisms of natural products for hepatorenal metabolic barrier injury.

Natural products possess unique advantages owing to their multi-component composition and multi-target action mode. They can precisely intervene in the specific pathological mechanisms underlying hepatorenal metabolic barrier injury. The intervention forms include herbal components, single Chinese medicinal herbs and Chinese medicinal prescriptions. These forms complement each other in intervening in hepatorenal injury and construct a targeted hepatorenal protection network (Figure 4; Tables 3–5).

**TABLE 3:** Intervention of herbal components on hepatorenal metabolic barriers injury.

Toxicity type	Compound category	Herbal components	Experimental models	ModelsTarget organs	Core mechanisms	Up-regulating targets	Suppression targets	References
Antibiotic-induced (GM)	Polyphenols	Resveratrol (RES), Sinapic acid (SA)	Albino mice, Wistar rat	Kidney	Alleviate epithelial-mesenchymal transition by inhibiting oxidative stress and regulating the TGF-β/Smad signaling pathway, downregulate renal oxidative/nitrosative stress, inflammation and apoptosis levels, and mitigate renal structural and functional damage	GSH, CAT, E-cadherin, NP-SH, IκBα, Bcl-2	p-Smad2, MDA, TGF-β1, α-SMA, Caspase 3, Bax, TNF-α, IL-6, NF-Κb p65, NF-Κb-DNA, MPO, γ-GGT, NO, Cleaved caspase-3, Nuclear NF-Κb	Beshay et al. (2020), Ansari et al. (2016)
	Polysaccharides	Polygonatum sibiricum polysaccharides (PSP)	Sprague Dawley rat	Kidney	Downregulate NGAL/KIM-1 Mrna expression, inhibit the p38 MAPK/ATF2 signaling pathway, reduce inflammatory cytokine levels, and exert renoprotective effects against AKI.	-	NGAL, TNF-α, P38 MAPK, IL-1β, KIM-1, IL-6	Han et al. (2020)
	Alkaloids	Palmatine	Sprague-Dawley rat	Liver, kidney	Inhibit OS and cellular apoptosis, and attenuate hepatotoxicity as well as nephrotoxicity	GSH	MDA	Khaksari et al. (2021)
	Organic acids	Oleanolic acid, Gymnemic acid (GA)	Albino Wistar rat	Kidney	Alleviate nephrotoxicity through antioxidant and diuretic effects, and protect renal tissues by reducing inflammation, OS and cellular necrosis	ETC complex-I, ETC complex-V, ETC complex-II, GSH, SOD, Catalase	MDA, TNF-α, IL-1β	Patil et al. (2010), Gumbar et al. (2023)
Drug-induced chemical (CDDP)	Polyphenols	Ellagic acid (EA), Paeoniflorin (Pae), Honokiol, Epicatechin gallate (ECG)	C57BL/6J mice, albino Wistar rat, HK2 cell	Liver, kidney	Alleviate hepatic peroxide-induced damage, alleviate and prevent AKI by regulating the Hsp90α1-Akt signaling pathway, maintain mitochondrial structural integrity and functional stability via SIRT3/AMPK-dependent mitochondrial dynamic remodeling, and downregulate the MAPK signaling pathway to inhibit OS.	GSH, GSH-Px, Bcl-2, Hsp90AA1, p-Akt, ATP, SIRT3, AMPK, ACC, LKB1, Drp1, SOD, CAT	GR, GST, LPO, KIM-1, TNF-α, NGAL, Caspase-3, Bax, MCP-1, IL-1β, IL-6, 4-HNE, Cytochrome C, Cleaved-caspase-3, NF-Κb p65	Goyal et al. (2019), Zhang et al. (2023), Mao et al. (2022), Malik et al. (2016)
	Terpenoids	Limonin, Lycopene (LYC), Crocin (CROC), Celastrol, Asiatic acid (AA), Madecassoside (MA), Curcumol (CUR)	C57BL/6J mice, L02 cell, Wistar rat, HK-2 cell, RTE cell, 4T1 cell, Hep3B cell, ES-2 cell	Liver, intestine, kidney	Modulate multiple signaling pathways, improve the hepatic metabolic microenvironment, inhibit liver injury and hepatotoxicity, exert anti-apoptotic, anti-inflammatory and renoprotective effects, and prevent and ameliorate AKI.	GPX4, NRF2, miRNA-9, miRNA-29, BAMBI m-RNA, Bcl-2, mtDNA, PCNA, PGC1-α, SOD, GSH-Px, Lrp2, Nlrp6, Mdh1, IL-10, HO-1, GPX, LC3A/LC3B, FTH1	P53, FADD, A-SAA, 4HNE, Bak, ACSL4, Inos, Cleaved caspase-7, COX2, Caspase-3, Fas, Bax, Bax/Bcl2, TLR4, MyD88, NF-Κb p50, Smad2, TGF-β1, MDA, IL-1β, MCP-1, IL-6, Phospho-TAK1, Cleaved caspase-3, NF-Κb p-p65, KIM-1, Phospho-IKKα/β, NGAL, Phospho-NF-Κb p65, TAK1, ROS, COX-2, TNF-α, NLRP3, p-P65, Cleaved-caspase1, HIF-1α, c-PARP, JNK, IL-12-α, Mmp14, Cxcl1, Cxcl2, P38, p-ERK, p-P38, F4/80, ERK, p-JNK, LPO, P62	Xiang et al. (2023), Yu et al. (2018a), Khedr et al. (2020), Yang et al. (2018), Shan et al. (2024), Jin et al. (2025)
​	Saponins	P. quinquefolium (PQS), Platycodon grandiflorum (PGS), Panax notoginseng saponins (PNS)	ICR mice, HK-2 cell	Kidney	Inhibit OS, inflammation, apoptosis and NF-Κb activation, regulate the PI3K/Akt apoptotic pathway, upregulate the HIF-1α/BNIP3 axis, inhibit the mitochondrial apoptotic pathway, and exert renoprotective effects against AKI.	GSH, SOD, Bcl-2, p-Akt, PI3K, p-PI3K, Akt, HIF-1α, BNIP3	Cleaved caspase-3, Nox4, Bax, COX-2, Cleaved Caspase-9, Inos, MDA, TNF-α, NF-Κb, IL-1β, CYP2E1, HO-1, p-NF-Κb, IKKα, IKKα/β, IκB, p-IKKα, p-IKKβ, Cyt C, Caspase-3, Bax/bcl-2	Ma et al. (2017), Zhang et al. (2018), Li et al. (2021)
	Polysaccharides	Trehalose, Flammulina Velutipes polysaccharides (FVPs)	C57BL/6J mice, HK2 cell	Kidney	Activate TFEB-mediated autophagy, preserve mitochondrial function, regulate gut microbiota, promote SCFA production, modulate renal metabolism, inhibit ferroptosis, and alleviate renal injury	Bcl-2, Parkin, P62, ATP5α, Sirt3, Ndufs4, Pink1, TFEB, Atg5, Becn1, Ctsb, Lamp, Lc3b, LC3 II, GSH, GPX-4, SLC7A11	Bax, LC3 I, TNF-α, IL-6	Zhu et al. (2020), Tu et al. (2025)
	Alkaloids	Leonurine, Sinomenine, Sanguinarine (SANG)	C57BL/6J mice, HK-2 cell, BALB/Cn mice	Kidney	Inhibit ferroptosis, activate Nrf2, target related signaling pathways to suppress OS, inflammation and apoptosis, reduce DNA damage and excessive autophagy, and protect against and alleviate CDDP-induced AKI.	NRF-2, NQO1, HO-1, SOD, GPX4, Xct, GSH, Bcl-2, Sirt6	TH-1, FTL, TFR, MDA, FTH-1, Fe, TNF-α, Akt, STAT3, NF-Κb, ERK1/2, p-JNK1/2, FOXO3a, NGAL, KIM-1, p-STAT3, HO-1, Cleaved caspase-8, 4-HNE, p-FOXO3α, 3-NT, P21, Bax, Noxa, Cleaved caspase-3, Cleavage of PARP1, p-ERK1/2, P38, Adiponectin, Complex I, TGF-β 1, Complex III, Cystatin c, TBARS, ROS, IL-1β, GSH, SOD, NO, Complex II, Complex IV, Beclin-1, LC3B, IL-6	Hu et al. (2022), Potočnjak et al. (2023), Zaaba et al. (2025)
​	Flavonoids	Luteolin, Tangeretin, Scutellarin	C57BL/6 mice, Wistar rat, HCT-116 cell, Hep3B cell	Kidney	Downregulate the p53 apoptotic pathway, inhibit NF-Κb activation, activate autophagy, enhance antioxidant defense, reduce apoptosis and inflammation, and ameliorate nephrotoxicity to protect renal tissues	GSH, Bcl-Xl, SOD, Catalase, Bcl-2, GPx, IL-10, LC3-II/LC3-I, ATG7	P53, Bax, Caspase-3, PUMA-α, Phospho-p53, Cleaved caspase-3, MDA, NO, Nrf-2, TNF-α, Cleaved PARP, Bax/p53, Bax/Bcl-2, P38, P62, IL-6, JNK, ERK, p-STAT3	Wang et al. (2024g), Arab et al. (2016), Sun et al. (2019a)
	Organic acids	Protocatechuic acid (Proto), Vanilic acid, p-Coumaric acid (PCA), Sinapic acid (SA)	Albino mice, Wistar rat	Liver, kidney	Neutralize oxidative stress, suppress inflammation, inhibit apoptosis, upregulate antioxidant enzymes and reduce oxidative markers, regulate Nrf2/HO-1 and inhibit NF-Κb activation, ameliorate and prevent hepatorenal injury	GSH, SOD, QLL, Caspase-3 V2-L, Caspase-3 (-vepop %), CAT, GPx, IL-10, Nrf2, HO-1, Bcl-2	Caspase-3, Bax, MDA, NO, Inos, NF-Κb p65, IL-6, TNF-α, QLR, QUL, QUR, Caspase-3 V2-R, Caspase-3 (+vepop %), TBARS, NF-Κb, IL-2, MPO	Sun et al. (2019a), Sindhu et al. (2015), Ekinci Akdemir et al. (2017), Ansari (2017)
Non-steroidal drug-induced (APAP)	Polyphenols	Pentagalloylglucose (PGG), Epigallocatechin-3-gallate (EGCG), Guavinoside B (GUB), Rosmarinic acid (RA), Genista quadriflora Munby (Gq)+Teucrium polium geyrii Maire (Tp), Chlorogenic acid (CGA), 4-hydroxyphenylacetic acid (4-HPA), Resveratrol (RSV)	Sprague-Dawley rat, HepG2 cell, Kunming mice, Wistar rat, SPF ICR mice, L02 cell, Mice, Male C57Bl/6 mice, primary mouse hepatocyte	Liver	Regulate the Nrf2, JNK and other signaling pathways, inhibit the Cgas-STING and NEK7-NLRP3 pathways, block Nrf2-Keap1 binding, reduce OS and toxic accumulation, scavenge peroxynitrite, prevent mitochondrial damage, and protect against and ameliorate liver injury	SOD, GSH, Bcl-2, LC3BII/LC3BI, NQO1, GCLC, GSH-Px, Nrf2, HO-1, GPx, GR, GST, NAD(P)H, P62, CONT, APAP, CAT, SOD1, SOD2, Mt-1	IFN-β, IL-6, p-IRF3, p-STING, IRF3, TBK1, MDA, TNF-α, STING, CXCL10, ISG15, Sulfotransferase, OATP1A1, CYP1A2, CYP2E1, CYP3A, UDP-glucuronosyltransferase, GSH peroxidase, NAD(P)H quinone 1 oxidoreductase, ROS, p-JNK, IL-1β, IL-18, NEK7, NLRP3, ASC, Caspase-1, TBARS, GSTpi, PP2A-A, PP5, Keap1, ERK1/2, 3-NT, GSSG, Catalase, MT-2, LDH, AIF, EndoG, Bax	Zheng et al. (2024), Yao et al. (2019), Li et al. (2020), Yao et al. (2022), Baali et al. (2016), Wei et al. (2018), Zhao et al. (2018a), Du et al. (2015)
​	Terpenoids	Rosmarinic acid (RA), Tanshinone IIA (Tan IIA)	C57BL/6J mice, Nrf2^+^/^+^ mice, HK-2 cell, L02 cell	Kidney	Antioxidize via pathway, upregulate pathway to promote toxin clearance, ameliorate liver injury and prevent its nephrotoxicity	SOD1, Nrf2, HO-1, GPx4, SLC7A11	MDA, SOD, RACK1, TNF-α, Nrf2, Mrps, Fe^2+^, TFR1, Fe^3+^	Yu et al. (2021), Zhang et al. (2022)
	Saponins	Radix Bupleuri (RB), Jujuboside B, Ginsenoside Rk3, Notoginsenoside Fc (Fc)	C57BL/6J mice, HepG2 cell, HEK293T cell, ICR mice	Liver, kidney	Inhibit complex assembly and autophagy-lysosome fusion, regulate related pathways, reduce oxidation, enhance antioxidants, inhibit inflammation, and improve liver injury as well as AKI renal tubular and mitochondrial damage	LAMP1, TFEB, p-ULK1 (Ser757), p-Mtor, ATG5, GSH, CAT, Bcl-2/Bax, SOD, SOD1, SOD2, Bcl-2, HO-1, Nrf2, NQO1, Cytosolic/Nuclear Nrf2, Beclin-1, ATG7, ATG12, SIRT3	STX17, SNAP29, VAMP8, Rab7a, TOM20, LC3-II/LC3-I, P62, CYP2E1, IL-6, IFN-β, MDA, TNF-α, NOX2, COX2, Bax, Cleaved-caspase-3, STING, Caspase-3, p-IRF3, p-P65, Keap1, Caspase-1, NLRP3, ROS, TLR4, Cytosolic P62, DAPI, LC3, IL-1β, Cystatin C, Ac-SOD2	Wang et al. (2024f), Wei et al. (2022a), Fan et al. (2025), Qu et al. (2021)
	Polysaccharides	Broussonetia papyrifera polysaccharide (BPP), Ganoderma lucidum polysaccharides (GLPs), Smilax china L. polysaccharide (SCLP)	Kunming mice, ICR mice, BALB/c mice, AML12 cell	Liver	Activate the Nrf2 pathway, inhibit toxic metabolism, regulate apoptosis, enhance antioxidant capacity, and protect against ALI.	GSH, SOD, t-SOD, Nrf2, Nrf2-ARE, GSH-Px, NQO1, GCLc, GCLm, HO-1, SULT1A1, UGT, CYP2E1, Bcl-2	MDA, Caspase-3, IL-6, Bax, Caspase-8, IL-10, Caspase-9, TNF-α, ROS, MPO	Zhang et al. (2024b), Xu et al. (2022b), Wang et al. (2022c)
	Alkaloids	Rutaecarpine, (+)-clausenamide, Berberine (BBR)	ICR mice, C57BL/6J mice, Hepa RG cell	Liver	Upregulate the Nrf2 antioxidant pathway, block its ubiquitination, inhibit oxidative stress, inflammation, necrosis and ferroptosis, and ameliorate and prevent liver injury and hepatotoxicity	GSH, IκBα, NRF2, GCLC, HO-1, NQO1, SLC7A11, GPX4, NADPH, GSTA1, GSTM2, TXNRD1, GCLM, Mn-SOD, SULT1A1, UGT1A1, UGT1A6, JNK, P65	MDA, TNF-α, CYP2E1, IL-1β, Phospho-IκBα, Keap1, Phospho-JNK1/2, NF-Κb p65, Phospho-NF-Κb p65, IL-6, Ptgs2, GLUT1, MPO, HMGB1, MCP-1, Cleaved caspase-1	Choi et al. (2021), Wang et al. (2020a), Zhao et al. (2018b)
	Flavonoids	Hinokiflavone (HF), Apigenin, Oroxyloside	C57BL/6J mice, HepG2 cell, Kunming mice, L02 cell	Liver	Target pathways to inhibit apoptosis and cell death, increase hepatic GR activity and GSH content, suppress JNK-related apoptosis and necroptosis, protect against and antagonize liver injury	JC-1, GSH, SOD, Bcl-2, SIX4, p-Akt, p-Stat3, GSH-Px, GR, GST	ROS, MDA, P53, Bax, NLRP3, ACS, IL-1β, GSDMD-N, Cleaved caspase-1, STAT3, CYP2E1, CYP1A2, IL-6, PARP, Caspase-9, JNK, RIPK3, MLKL, Caspase-3	Liu et al. (2024b), Yang et al. (2013), Liao et al. (2020)
​	Organic acids	Glycyrrhizic acid (GA), Chlorogenic acid (CGA)	C57BL/6J mice, AML12 cell, ICR mice	Liver	Activate the β-catenin pathway to promote hepatocyte proliferation, block apoptotic and OS-induced damage, and alleviate ALI.	Β-catenin, PCNA, Cyclin D1, CDK4, CV, PV, EdU, GCL, Trx1/2, TrxR1/2, Pro-caspase-3, Pro-caspase-7, GSH, GR, GPx, GST, GCLC, GCLM	Cleaved-caspase-3, MEK1/2, Cleaved-caspase-7, Phosphorylation ASK1, Phosphorylation ERK1/2, MKK3/6, Phosphorylation JNK, Phosphorylation p38, c-Raf, MKK4	Cao et al. (2025), Ji et al. (2013)
Heavy metal-induced (Mn,Cd,Pb,Cr,Zn,Cu,uranium, mercury chloride)	Polyphenols	(−)-epigallocatechin gallate (EGCG), Resveratrol	Juvenile carp, BALB/c mice, RAW 264.7 cell	Kidney, liver	Regulate the TRPM2-NLRP3-TNF-α-JNK pathway to alleviate renal injury, ameliorate mitochondrial abnormalities and reverse associated autophagic disorders, thereby exerting hepatorenal protective effects	GSH, Cu-Zn SOD, GSH-Px, NXR, VDAC1, CAT, Cyt C, Sirt3, T-SOD, GST	H_2_O_2_, Trpm2, Trpm6, SOD, CAT, GPx, Gpx1b, Gpx4a, MDA, Sirt1, PGC-1α, Nrf1, TFAM	Zhou et al. (2023), Zhang et al. (2020b)
	Terpenoids	Oleanolic acid, Carvacrol (CRV), Stevioside	C57BL/6 mice, Sprague Dawley rats, Thinlip mullet	Liver, kidney	Inhibit ROS and toxicant overload, counteract hepatorenal oxidative damage and apoptosis, exert multidimensional regulation to reduce toxicity, and alleviate hepatic ferroptosis and hepatotoxicity	CAT, GSH, GPx, SOD, Bcl-2	MDA, GSH, CAT, GPx, Bax, P53, Caspase-6, Caspase-9, Caspase-3, NF-Κb, MAPK14, Bcl-3, Inos, PGE2, 8-OhdG, IL-1β	Kandemir et al. (2021), Ouyang et al. (2023), Shehata et al. (2024)
	Saponins	Ginsenoside Rg1, Saikosaponin a (Ssa)	C57BL/6J mice, AML-12 cell, Fish	Liver, kidney	Activate PPAR-γ and regulate the TLR4/MyD88/MAPK pathway to protect the liver, inhibit the NF-Κb/Nrf2 axis and block inflammation and OS to antagonize renal injury	SOD, GSH, CAT, PPAR-γ, HO-1, Nrf2	MDA, ROS, IL-6, Inos, TNF-α, IL-12, NO, MPO, Keap1, IL-1β	Zhao et al. (2025), Song et al. (2021)
​	Polysaccharides	Emblica officinalis polysaccharide (EOP), Sagittaria sagittifolia polysaccharide (SSP), Phormidim versicolor NCC466 crude polysaccharides (CFv-PS), Flammulina velutipes residues polysaccharide (FVRP), Sparassis latifolia polysaccharide, Lycium barbarum polysaccharide (LBP), Polygonatum kingianum polysaccharides (PKP)	BALB/C mice, Kunming mice, L02 cell, Wistar rat, HepG2 cell, ICR mice, HK-2 cell	Live, kidney, brain	Activate Nrf2 and its related signaling pathways, inhibit OS, apoptosis and inflammation, regulate metabolism, autophagy and gut microbiota, protect against heavy metal-induced hepatorenal injury, and alleviate cytotoxicity	Nrf2, HO-1, Bcl-2, IL-10, SOD, T-SOD, GSH, T-SH, NQO1, CAT, GPx, ascorbic acid, GSK3β, TGF-β, LPIN, DGAT, IL-4, GST, MMP, ROS	Bax, IL-1, TNF-α, NAD(P)H, Caspase-3, Active caspase-3, Bcl-Xl, MDA, ROS, ALAT, DNA, Protein carbonyl, MT, ASAT, Bilirubin, IL-6, IL-18, IL-1β, Cytochrome c, Caspase-9, active caspase-9, Beclin-1, LC3-I, LC3-II, Atg5, P62, LAMP2, NO, Fyn	Wang et al. (2023c), Liu et al. (2022c), Belhaj et al. (2018), Liu et al. (2023d), Lu et al. (2024), Xie et al. (2021), Li et al. (2022b)
	Flavonoids	Luteolin (LUT), Vitexin	Su poultry green shell grass chicken, albino rat	Liver, intestine, kidney	Stabilize liver-gut homeostasis, regulate microbiota, resist oxidation, inflammation and apoptosis, activate the AMPK/SIRT1/FOXO1 pathway, and alleviate Cd-induced hepatorenal injury and metabolic disorders	GSH, T-AOC, T-SOD, CAT, TP, ALB, A/G, GSR, GST, GLUT8, GSK-3β, ATGL, CPT1, PPAR, MCAD, LCAD, GSH-Px, GCLC, GCLM, SIRT1, p-AMPK	LPS, MDA, BA, GLB, γ-GT, ROS, NF-Κb, TNF-α, IL-1β, IL-6, COX-2, Bax, Caspase-9, Caspase-3, GLUT1, PK, PFK, PCK2, HK2, FAS, SREBP1, CPT2, IL-8, FOXO1, p-SIRT1	Wang et al. (2024g), Zou et al. (2024), Noor et al. (2022)
Organic solvent-induced (CCl_4_)	Polyphenols	Oxyresveratrol (OXY), Resveratrol, Apple polyphenols (AP), Seedless black Vitis vinifera polyphenols	C57BL/6 mice, HepG2 cell, BALB/c mice, RAW264.7 cell, Swiss-Kunming mice	Liver, kidney, lung, brain, spleen	Activate Nrf2 to protect hepatocytes from OS/mitochondrial dysfunction, upregulate IL-10 to modulate microenvironment and promote macrophage polarization, inhibit proinflammatory cytokines and activate antioxidant system to preserve liver function, block ROS/NF-Κb pathway for multi-organ protection	PARP, Bcl-Xl, Nrf2, ARE, GSTA2, GCLC, p-Nrf2, GSH, p-ERK, HO-1, Pro-caspase-3, IL-10, IL-4, Mrc1, Mrc2, CD163, Arg1, CD206, SOD, CAT, GPx	DCFH-DA, nitrotyrosine, 4-HNE, Inos, TNF-α, MCP1, IFN-γ, NO, ROS, TBARS, MPO, COX-2, IL-8	Choi et al. (2016), Yu et al. (2019), Wang et al. (2016), Habashy et al. (2021)
	Terpenoids	Catalpol, Glycyrrhizic acid (GA), Ursolic acid (UA), Ganoderic acid A (GAA)	C57BL/6J mice, LX-2 cell, HepG2 cell, BRL-3A cell, ICR mice	Liver, kidney	Inhibit the EphA2/FAK/Src pathway to block HSC aerobic glycolysis and treat hepatic fibrosis, target AKR7A2 and inhibit the NOX4/ROS pathway to reverse hepatic fibrosis, regulate the Trx/TrxR pathway for renal immune protection	SOD, CAT, GSH, MMP-1, TrxR, Trx, GPx	TNF, IL-6, IL-1β, IL-18, α-SMA, Collagen I, HK2, PFKFB3, PKM2, ENO1, p-EphA2, HA, PIIIP, Collagen IV, LN, MDA, TIMP-1, NOX4, RhoA, ROCK1, NF-Κb, Smad3, p-Smad3, p-STAT3, TGF-β1, p-JAK2	Wan et al. (2020), Zhang et al. (2024c), Wang et al. (2024h), Ma et al. (2021)
	Saponins	Red ginseng saponins, Soyasaponin Bb, Ginsenoside Rk1	Sprague Dawley rat, HSC-LX2 cell, C57BL/6 mice, HepG2 cell	Liver	Inhibit P450 enzyme to improve hepatic microsomal lipid peroxidation for liver protection regulate TGF-β1/α-SMA pathway to reduce inflammation and resist hepatic fibrosis promote apoptosis via AMPK/Mtor pathway to inhibit primary liver cancer	SOD, Bcl-2, ATG5, Beclin-1, LC3, p-AMPK/AMPK	MDA, TGF-β1, α-SMA, IL-6, IL-1β, Cleaved-caspase3, Cleaved-PARP, P62, p-Mtor/Mtor	Kim et al. (1997), Lin et al. (2025), Wu et al. (2024b)
​	Polysaccharides	Angelica sinensis polysaccharide (ASP), Anoectochilus roxburghii polysaccharides (ARP), Cyclocarya paliurus polysaccharides (CP)	C57BL/6J mice, hepatic stellate cell, Kunming mice, NCTC-1469 cell	Liver, kidney	Regulate the IL-22/STAT3 pathway to inhibit HSC activation and alleviate hepatic fibrosis, enhance hepatic antioxidant enzyme activity to suppress lipid oxidative damage, and exert antioxidant effects to ameliorate hepatorenal OS.	IL-22, p-STAT3, GSH, T-AOC, GSH-Px	ALT, α-SMA, Collagen I, MMP2, TIMP1, MDA, CYP2E1	Wang et al. (2020b), Yang et al. (2017), Wu et al. (2020)
	Flavonoids	Nobiletin (NOB), Carlinoside, Eupatilin	C57/BL6J mice, L02 cell, albino rat, HepG2 cell, LX-2 cell	Liver	Induce autophagy and regulate the Hippo/YAP pathway to protect hepatocytes, activate Nrf2 and UGT1A1 to ameliorate hyperbilirubinemia, inhibit the β-catenin pathway and EMT to alleviate hepatic fibrosis	LC3, Beclin-1, MMP2, CCN1, CCN2, UGT1A1, Nrf2	α-SMA, NLRP3, IL-18, IL-1β, YAP, TEAD2, P62, N-cadherin, Vimentin, TIMP1, TIMP2, HO-1, COL1α1, CyclinB1, CyclinD1, CDK6, c-Myc, PAI-1, β-catenin	Hao et al. (2024), Kundu et al. (2011), Hu et al. (2023)
	Organic acids	Lithospermic acid (LA), Salvianolic acid B (Sal B)	C57BL/6 mice, SD rat, HSC cell, Kupffer cell, LO2 cell	Liver	Regulate Piezo1 pathway to inhibit hepatic fibrosis, reduce NOX/ROS, promote hepatocyte proliferation, inhibit HSC and KC, upregulate Ecm1 and inhibit ferroptosis to alleviate hepatic fibrosis	GSH, SOD, GSH-Px, Nrf2, ACSL4, MMP-1, Bcl-2, XIAP, Fth1, Ftl, Xct, GPx4	Collagen I, IL-1β, Collagen III, α-SMA, TGF-β, NOS2, Notch1, Jag1, IL-6, DLL4, TNF-α, Hes1, Hey1, Hey2, GPX4, 4-HNE, NOX1, MDA, P67phox, Caspase9, TGF-β1, TIMP-1, NOX4, NOX2, CD95, P22phox, ROS, Gadd45β, Bax, CD95L, P47phox, Rac1, Caspase3, Acsl4, Ptgs2, Tfrc	Luo et al. (2024), Gan et al. (2018), Fu et al. (2024)
Biological toxin-induced (AFB1,α-AMA)	Polyphenols	Curcumin, Quercetin (QUE), Oxidised tea polyphenols, Resveratrol	Albino rat, T-antigen-transduced foetal hepatocyte-derived cell, SD rat, Caco-2 cell, duck	Liver	Regulate the NLRP3/Nrf2 pathways, stabilize lipid homeostasis, inhibit pyroptosis and oxidative stress, block AFB1 absorption and metabolism, modulate related enzymes and signaling pathways, and alleviate hepatotoxicity	Nrf2, CAT, NQO1, GSH, GSS, HO-1, GCLC, GCLM, GST, GSTA1, GPX, SOD, SOD1, T-AOC, CYP1A1, CYP1B1, AHR, AHRR, ARNT, Sirt1, Bcl-2	IL-1β, ROS, NO, MPO, NF-Κb, Inos, TNF-α, COX-2, IL-18, GSTA1, CYP3A28, SOD2, GPX1, Keap1, MDA, H_2_O_2_, CYP450, CYP1A1, CYP1A4, CYP3A4, IL-16, IL-10, Caspase3, Caspase9, Bax	Wang et al. (2022a), Pauletto et al. (2023), Lu et al. (2017), Qiao et al. (2023), Liu et al. (2021)
	Terpenoids	Beta-caryophyllene (BCP), Lupeol, Lycopene, Cannabidiol, βeta-carotene (Βc)	Wistar rat, albino rat, Kunming mice, C57BL/6J mice, L02 cell, Sprague Dawley rat	Liver, kidney	Neutralize OS and suppress inflammation, enhance antioxidant capacity, activate the Nrf2 pathway, regulate autophagy and the NOS/AR pathway, and protect against hepatorenal injury and nephrotoxicity	Nrf2, IKKβ, CAT, GPx, GR, G6PD, GSH, SOD1, GCLC, GCLM, GSS, Bcl-2, GST, NOS	Keap1, NF-Κb, IL-1β, MDA, H_2_O_2_, Bax, Cyt-C, Cleaved caspase-3, MPO, AR	Da Silveira et al. (2021), Preetha et al. (2006), Karaca et al. (2021), Yu et al. (2018b), Wang et al. (2023d), Gezer et al. (2024)
	Polysaccharides	Salvia miltiorrhiza polysaccharide (SMP)	New Zealand meat rabbit	Liver	Activate Nrf2/HO-1, inhibit mitochondrial apoptotic pathway, alleviate liver injury	Nrf2, HO-1, Bcl-2	MDA, TNF-α, 4-HNE, ROS, IL-1β, Caspase	Zhang et al. (2024d)

**TABLE 4:** Intervention of single Chinese medicinal herbs on hepatorenal metabolic barriers injury.

Toxicity type	Single Chinese medicinal herbs	Experimental models	Target organs	Core mechanisms	Up-regulating targets	Suppression targets	References
Antibiotic-induced (GM)	*Fagonia olivieri*, Riceberry bran extract, *Sonchus asper*, *Malva sylvestris* extract, Red ginseng extract, *Rhizoma smilacis glabrae*	SD rat	Liver, kidney	Protect against hepatorenal injury via antioxidation, anti-inflammation, anti-apoptosis, OS regulation, caspase-3 inhibition, and alleviation of nephrotoxicity-induced hepatic damage	Catalase, SOD, GR, GSH, DNA, SOD2, Nrf2, BCL-Xl, CAT, POD, FRAP, Bcl-2	GSH-Px, TBARS, NF-Κb, MDA, HO-1, COX2, Bax, Inos, Cleaved Caspase-3, TNF-α, Caspase-3, ICAM-1, ROS, NOX, Cytochrome c	Liu et al. (2017b), Rashid and Khan (2017), Arjinajarn et al. (2016), Khan et al. (2011), Mohamadi Yarijani et al. (2019), Shin et al. (2014)
Drug-induced chemical (CDDP)	*Astragali Radix*, *Filipendula ulmaria*, Pomegranate extract, *Schisandra chinensis* bee pollen extract, *Sonchus cornutus*, *Capparis spinosa* leaves	ICR mice, Albino Wistar rat, SD rat	Liver, kidney	Alleviate hepatorenal damage via antioxidant, anti-inflammatory and anti-apoptotic effects, regulation of OS, serum metabonomics and intestinal flora, H_2_O_2_ scavenging, and lipid peroxidation antagonism	CAT, SOD, GSH, IL-10, Nrf2, Bcl-2, GSH/GSSG, Catalase, GPx	TBARS, Caspase 9/3, IL-1β, MDA, Inos, NF-Κb, P53, Bax, Cytochrome C, IL-6	Wang et al. (2022b), Huang et al. (2017), Katanić et al. (2017), Bakır et al. (2015), Elhady et al. (2022), Tlili et al. (2017)
Non-steroidal drug-induced (APAP)	)Zhishi, *Paeonia lactiflora* Pall., *Aloe vera*, *Cardiospermum halicacabum*, Cinnamon, *Crepis rueppellii* Leaf	SD rat, C57BL/6 mice, ICR mice, Wistar rat, Balb/c mice, Guinea pig	Liver, kidney	Alleviate hepatorenal injury by reducing oxidative stress, restoring hepatic GSH levels, improving renal function and histopathology, enhancing antioxidant activity, and ameliorating renal leukocyte infiltration and necrosis	GSH, MMK7, Nrf2, HO-1, GPX4, Bcl-2, SOD, IL-12, IL-18, TAC	P53, Bax, Caspase-3, p-MMK7, p-AMPK7, JNK1, p-JNK1, MDA, CYP3A4, Fe^2+^, Bad, CytoC, p-PKC, p-ERK1/2, P90RSK, SGOT, SGPT, TOS	Shu et al. (2020), Li et al. (2024b), Werawatganon et al. (2014), Parameshappa et al. (2012), Hussain et al. (2019), Kaidama et al. (2025)
Heavy metal-induced (Hg/Cd/Pb)	Raw garlic, *Aronia melanocarpa* berry extract, *Panax ginseng*, *Withania somnifera* root extract, *Coriandrum sativum*, *Dendropanax morbifera* stem extract	Wistar rat, Swiss albino mice, ICR mice, SD rat	Liver, kidney, femur, blood, brain	Inhibition of toxicant deposition, regulation of oxidative balance, reduction of LPO and liver enzymes, and elevation of antioxidant enzymes	GPx, SOD, CAT, GR, GST, TSH, GSH, GSH/GSSG, TAS, TOS	GSSG, H_2_O_2_, MPO, XOD, OSI, MDA, SGOT, SGPT, LPO	Mężyńska et al. (2018), Nwokocha et al. (2012), Shukla and Kumar (2009), Chaurasia et al. (2000), Aga et al. (2001), Kim et al. (2014)
Organic solvent-induced (CCl_4_)	*Trigonella foenum graecum*, *Mori fructus* aqueous extract, *Lens culinaris* sprout extract, Dandelion extract, *Heliconia rostrata* rhizome, *Suaeda vermiculata*	Wistar rat, ICR mice, Grouper, Swiss albino rat, SD rat	Liver, kidney	Protect against hepatorenal injury by stimulating β-oxidation, reducing lipid accumulation and OS complications, elevating antioxidant levels, downregulating inflammatory responses, and enhancing immunity	GSH, SOD, CAT, GR, CPT-1, MHC-2, TLR3, Caspase 8/9, IL-10	MDA, CAT, GPX, Keap1, G6PD, PPAR-α, IL-8, Caspase 3	Wei et al. (2022b), Mbarki et al. (2017), Barakat et al. (2022), Sun et al. (2019b), Dhara et al. (2021), Mohammed et al. (2020)
Biological toxin-induced (AFB1)	*Radix Bupleuri* extract, *Penthorum chinense* extract, Sea buckthorn	Pekin-duck, Cobb broiler	Liver	Protect against liver injury via antioxidant and anti-apoptotic effects, reducing hepatic oxidative stress, reversing liver damage, and lowering hepatic toxicant concentration	T-AOC, SOD, CAT, Nrf2, HO-1, Bcl-2, Bcl-2/Bax, GSH, GSH-Px, IgA, IgG, IgM, COX-2	ROS, MDA, NQO1, Keap1, Caspase-9/3, Bax, Cleaved Caspase-3, P53, Bak	Feng et al. (2024), Nabi et al. (2022), Solcan et al. (2013)

**TABLE 5:** Intervention of Chinese medicinal prescriptions on hepatorenal metabolic barriers injury.

Toxicity type	Chinese medicinal prescriptions	Experimental models	Target organs	Core mechanisms	Up-regulating targets	Suppression targets	References
Drug-induced chemical (CDDP)	Huangqi-Danshen Decoction, Yi-Qi-Jian-Pi-Xiao-Yu Formula, Yi-Shen-Xie-Zhuo Formula	C57BL/6 mice	Kidney	Suppress renal apoptosis, inflammation and oxidative stress, inhibit ferroptosis via STING-NCOA4-mediated ferritinophagy, and exert renoprotective effects through cGAS/STING pathway-mediated anti-inflammatory and anti-apoptotic actions	NAM, NAAD, NAD+, NAMPT, QPRT, NMNAT1, LY6C, GPX4, Fpn, BCL-2	Bax, P53, p-P53, F4/80, 4-HNE, 8-OHdG, Cleaved caspase-3, QA, QA/Tryptophan, MDA, NGAL, Ccl2, CD68, LY6G, IL-1β, α-SMA, TGF-β1, Col3α1, IL-6, MMP-9, ALOX15, PTGS2, Fe (Chrysopoulou and Rinschen, 2024)+, TFR1, FTH, TNF-α, FTL, NCOA4, P62, LC3, STING, ALOX15, KIM-1, CXCL10, Havcr1, ICAM1, MCP-1, IL-8, cGAS, TBK1, IRF3	Liu et al. (2023c), Zhu et al. (2024), Qi et al. (2023)
Non-steroidal drug-induced (APAP)	Shaoyao-Gancao Decoction	C57BL/6 mice	Liver	Effectively attenuate hepatotoxicity by promoting autophagy and mitophagy	JNK, ATG4a, Atg3, PIK3C3, PINK, PARKIN, ULK2	p-JNK, IL-1β, MDA, F4/80, MPO, APAP-CYS, P62, TNF-α, CYP2E1, IL-6	Wu et al. (2024c)
Heavy metal-induced (Pb)	Xiao-Chai-Hu Decoction	ICR mice	Liver	Alleviate hepatic injury by modulating endocrine function	-	Esr1, Hsp90aa1	Jia et al. (2021)
Organic solvent-induced (CCl_4_)	ErTao Decoction, Qijia Rougan Decoction, Xiaoyaosan Decoction, Jiawei Taohe Chengqi Decoction, Shenlian Decoction, Xuefuzhuyu Decoction, Baihe Wuyao Decoction (BWD), Dahuang-Wumei Decoction, Xiayuxue Decoction, SiNiSan, Yi Guan Jian Decoction, Tao-Hong-Si-Wu Decoction (THSWD)	C57BL/6J mice, Sprague–Dawley rats, Wistar rats, LX-2 cells, Kunming mice	Liver	Regulate multiple signaling pathways, inhibit macrophage and HSCs activation and hepatic fibrosis, exert anti-inflammatory and antioxidant effects, and promote hepatic stem cell differentiation and liver regeneration	CD163, Cxcl12, M1/M2, IL-1, Alb, DDAH1, MMP2, MMP9, MMP12, SOD, NOR, ZO-1, Claudin5, Occludin, Wnt, DVL2, p-GSK3β, β-catenin, Bcl-2, ERK1/2, p-P38, MMP-13, TIMP-2, Wnt-1/-3α, LRP-5/-6, FZD5, ARG-1, mMGL2, IL-10, p-AMPK/AMPK, VEGF, VEGFR-2, HIF-1α, p-Akt, p-FoxO3a, DAPI, p-ATF2, CUGBP1, Serpinf2, Serpina1d, Serpina1α, Serpind1, Serpina1e, KNG1, PIg, Chb, Cfh, C5, C5a-C5aR, F4/80CD11b, CK19, EpCAM, Flt-1, KDR, Akt, pAkt	Col I, α-SMA, TGF-β1, Col III, p-SMAD2, TGF-βR1, PC III, LN, Col1a1, TIMP1, Smad3, Collagen I, TGF-β3, Cxcl1, TGF-β1i1, TGF-β2, AOD, HA, IV-C, HYP, MDA, iNOS, IL-1β, IL-11, TNF-α, FN, GFAP, IL-6, APC, AXN1, Bax, Caspase-3, Cytochrome C, JNK, ERK, p-JNK, Cleaved caspase-3, TIMP-1, OV6, IRF3, IRF5, NF-κB, IRF8, FZD2, SOX9, Wnt-4/-5A/-5B, CD68, STAT1, OCS3, FZD-2/-4/-6	Jia et al. (2025b), Zhang et al. (2020a), Xu et al. (2022a), Xu et al. (2021b), Chen et al. (2022), Zhou et al. (2021), Ye et al. (2024), Li et al. (2024c), Zhou et al. (2014), Chen et al. (2020), Liu et al. (2024c), Mu et al. (2009), Xi et al. (2016)

### 6.1 Intervention for hepatic metabolic barrier injury

Natural products are characterized by diverse components, broad targeting profiles and low toxicity and side effects. Thus, they have become an important research direction for the intervention of hepatic metabolic barrier injury. Specifically, herbal components can accurately target the key pathological links of hepatic metabolic barrier injury, while single Chinese medicinal herbs and Chinese medicinal prescriptions realize multi-dimensional and systematic barrier protection. The two categories act synergistically, thus forming the core system of natural products for intervening hepatic metabolic barrier injury.

#### 6.1.1 Intervention with herbal components

Core herbal components including polyphenols, terpenoids and saponins exert specific ameliorative effects on key pathological abnormalities of hepatic metabolic barrier injury. These abnormalities mainly involve metabolic enzyme disorders, hepatic sinusoidal structural damage and cholestasis. They exert precise hepatoprotective effects through specific functional pathways. These components act as the core carriers for natural products in intervening hepatic metabolic barrier injury.

The representative components of polyphenols are ellagic acid (EA), pentagalloyl glucose (PGG) and curcumin. These components can activate the Nrf2 antioxidant pathway, upregulate the expression of antioxidant enzymes such as SOD and GSH-Px, and protect the activity of the CYP450 metabolic enzyme system. They can also reduce the generation and accumulation of toxic intermediates like NAPQI during APAP metabolism (Goyal et al., 2019; Zheng et al., 2024; Wang Y. et al., 2022). Terpenoids are mainly used to prevent and treat liver metabolic disorder and hepatic fibrosis. Limonin, ursolic acid (UA) and β-caryophyllene (BCP) exert distinct hepatoprotective effects against chemotherapeutic DILI via their respective mechanisms. Specifically, limonin alleviates acute liver injury directly, UA acts by inhibiting ROS production and HSCs activation, and BCP reduces hepatotoxicity and retards hepatic fibrosis progression through antioxidant and anti-inflammatory pathways (Xiang et al., 2023; Da Silveira et al., 2021; Wan et al., 2020). Saponins focus on improving bile excretion disorder and inhibiting hepatocyte apoptosis. Jujuboside B, Ginsenoside Rg1 and other components can play a role by regulating transporter-related pathways. Restore the functions of transporters such as MRP2 and BSEP, and alleviate the liver injury caused by cholestasis (Wang H. F. et al., 2024; Zhao et al., 2025). In addition, polysaccharides exert hepatoprotective effects through dual pathways. They not only directly protect hepatocytes but also regulate the intestinal-hepatic axis (Zhang N. et al., 2024). Alkaloids, flavonoids and organic acids synergistically regulate OS, inflammation and apoptosis. Through multi-channel regulation, a multi-dimensional liver barrier protection network is formed.

#### 6.1.2 Intervention with single Chinese medicinal herbs and Chinese medicinal prescriptions

Single Chinese medicinal herbs and Chinese medicinal prescriptions have the advantages of multi-component and multi-target. Both of them can systematically intervene the damage of hepatic metabolic barrier from multiple links, and effectively make up for the limitation of single herbal component.

The protective effect of single Chinese medicinal herbs is different. Astragali Radix can regulate serum metabonomics and intestinal flora balance, and effectively alleviate CDDP-induced liver injury (Wang L. et al., 2022). Zhishi can inhibit the activity of p53 upregulated modulator of apoptosis (PUMA) protein induced by APAP and block the apoptosis pathway of hepatocytes. At the same time, it regulates the key targets of liver lipid metabolism and reduces chemical liver injury (Shu et al., 2020). Aronia melanocarpa berry extract can maintain the redox balance in the liver and block the onset and progression of OS. It exerts a protective effect against cadmium-induced liver injury through this mechanism (Mężyńska et al., 2018).

Chinese medicinal prescriptions follow the holistic concept of TCM and the principle of syndrome differentiation and treatment. They are featured by strong targeting and stable curative effects. Among them, Xiao-Chai-Hu decoction can regulate the expression of related genes and alleviate lead-induced liver injury (Jia et al., 2021). Ertao decoction inhibits inflammatory reaction and reduces collagen deposition through double action mechanism, thus reducing the degree of hepatic fibrosis (Jia H. et al., 2025). Classical compound prescriptions include Xiayuxue Decoction, SiNiSan and Yiguan Decoction. They exert protective effects on the hepatic metabolic barrier in an all-round manner through multiple mechanisms such as promoting autophagy, resisting OS and improving bile excretion. These prescriptions can also effectively alleviate various types of liver injury (Zhang D. et al., 2020; Xu W. et al., 2022; Xu Y. et al., 2021).

### 6.2 Interventions for renal metabolic barrier injury

Renal metabolic barrier injury involves multiple pathological disorders including glomerular filtration, renal tubular transport and renal interstitial homeostasis. This injury is the core pathological basis of renal damage induced by drugs and environmental toxicants. The damage repair and functional regulation of renal metabolic barrier need to rely on multi-target and multi-channel coordinated intervention strategies. Herbal components, single Chinese medicinal herbs and Chinese medicinal prescriptions have become important research directions of renal metabolic barrier injury intervention by virtue of their diversity of components and pleiotropic effects.

#### 6.2.1 Intervention with herbal components

All kinds of herbal components can accurately target the core injury targets of renal metabolic barrier, such as GFB, renal tubular transporter, renal interstitial inflammation, etc. Combined with the core functional characteristics of renal barrier “filtration-transport-interstitial stability,” renal protection can be achieved through multiple channels and multiple targets, covering major pathological conditions such as AKI, renal fibrosis and NS.

Polyphenols are represented by resveratrol and paeoniflorin. Resveratrol inhibits the epithelial-mesenchymal transition of renal tubular epithelial cells induced by gentamicin. This action maintains the redox balance of renal tissue and blocks the occurrence and development of renal fibrosis (Beshay et al., 2020). Paeoniflorin regulates the heat shock protein 90 alpha family class A member 1-protein kinase B (HSP90A1-AKT) pathway. This regulation inhibits CDDP-induced apoptosis of renal tubular epithelial cells, reduces the expression of proinflammatory factors and alleviates renal interstitial inflammation (Zhang et al., 2023). Terpenoids focus on improving renal tubular dysfunction and renal interstitial inflammation. Limonin, celastrol and carvacrol each exert renal protective effects via distinct mechanisms. They respectively antagonize inflammatory pathways, enhance antioxidant enzyme activity and inhibit ferroptosis (Xiang et al., 2023; Yu X. et al., 2018; Kandemir et al., 2021). In addition, saponins such as notoginsenoside Fc can repair glomerular podocyte injury and reduce renal tubular cell apoptosis (Wei M. et al., 2022). Polygonatum sibiricum polysaccharides and other polysaccharides can downregulate the expression of renal injury markers and inhibit inflammatory pathways to resist AKI (Han et al., 2020). Alkaloids such as leonurine can alleviate kidney injury by activating antioxidant pathway and inhibiting iron death (Hu et al., 2022). Flavonoids such as luteolin regulate intestinal flora, stabilize intestinal-renal axis and reduce renal interstitial inflammation (Wang X. et al., 2024).

#### 6.2.2 Intervention with single Chinese medicinal herbs and Chinese medicinal prescriptions

Single Chinese medicinal herbs and Chinese medicinal prescriptions focus on the structural repair of the renal metabolic barrier. They also regulate the function of the renal metabolic barrier. They improve drug- and environmental toxin-induced renal injury via the synergistic effect of multiple components. They are an important part of natural products' renal protection, and they are complementary to herbal components.

Among single Chinese medicinal herbs, Rhizoma smilacis glabrae (RSG) exerts a protective effect on gentamicin-induced renal injury. It achieves this by inhibiting caspase-3 activation and blocking oxidative stress-induced renal tubular cell apoptosis (Liu C. et al., 2017). Mori fructus aqueous extract enhances renal antioxidant capacity by activating the Nrf2 signaling pathway. It reduces CCl_4_-induced OS injury in renal tissue and renal interstitial inflammation. It creates a favorable microenvironment for the repair of renal tissue (Wei Y. Y. et al., 2022). Schisandra chinensis Bee Pollen Extract reduces the level of OS in renal tissue. It enhances the antioxidant, anti-inflammatory and anti-apoptotic capacities of the kidney. It alleviates CDDP-induced damage to renal structure and function (Huang et al., 2017).

Chinese medicinal prescriptions follow the principles of TCM and realizes multi-link systematic intervention on renal metabolic barrier injury. Huangqi-Danshen Decoction can effectively interfere with CDDP-induced AKI. It exerts this effect by regulating the nicotinamide adenine dinucleotide (NAD) biosynthesis pathway, increasing renal tissue NAD+ levels, inhibiting renal tubular cell apoptosis and renal interstitial inflammation (Liu X. et al., 2023). Yi-Qi-Jian-Pi-Xiao-Yu formula inhibits iron death of renal tubular epithelial cells through ferritin autophagy pathway mediated by stimulator of interferon genes–nuclear receptor coactivator 4 (STING-NCOA4), and relieves renal injury induced by CDDP (Zhu et al., 2024). In addition, classic compound formulas such as Yi-Shen-Xie-Zhuo Formula inhibit the inflammatory reaction and fibrosis process of renal interstitium by regulating the RAS and blocking the cyclic GMP-AMP synthase/stimulator of interferon genes (cGAS/STING) pathway. This exerts a significant renal protective effect (Qi et al., 2023).

### 6.3 Bioavailability bottleneck of natural products

Although natural products have a significant protective effect on the damage of hepatorenal metabolic barrier through multi-component and multi-target regulation, their clinical application is hindered by a significant bioavailability bottleneck. Curcumin, quercetin and similar natural products have shown good prospects for protecting liver and kidney in experimental research. However, their poor water solubility, rapid intestinal metabolism and extensive first-pass elimination lead to their low bioavailability and tissue concentration in liver and kidney less than (Feng et al., 2017; Mirza et al., 2023). These limitations limit its *in vivo* efficacy, and represent a key challenge in further transformation research against hepatorenal metabolic barrier damage.

## 7 Conclusion and discussion

This review systematically combs the structural and functional characteristics of hepatorenal metabolic barriers and the specific damage mechanisms of drugs and environmental toxicants to them. It also summarizes the targeted intervention strategies of natural products for these barrier injuries. The hepatic metabolic barrier is centered on the three-layer structure of “hepatic sinus-metabolizing enzyme-bile excretion”. Toxic damage mainly targets this barrier and exerts adverse effects on multiple key sites. These effects include the destruction of hepatic sinus endothelium, CYP450 enzyme system disorder and transporter-mediated cholestasis.

Renal metabolic barrier depends on the synergistic effect of “glomerular filtration barrier-renal tubule-renal interstitial blood vessels”. Its damage mostly targets podocyte dysfunction, OATs/OCTs transporter disorder, and interstitial inflammation and fibrosis. The difference between them in injury target and pathological process stems from the essential difference of organ metabolic function. With the unique advantages of multi-component and multi-target, natural products can accurately regulate the specific pathways of hepatorenal injury. For the liver, the intervention focuses on protecting the activity of metabolic enzymes and blocking the process of hepatic fibrosis. For the kidney, it centers on repairing the filtration membrane and improving renal tubular function.

Although research on the damage mechanisms of hepatorenal metabolic barriers and natural product interventions has made progress, several application bottlenecks still exist. Future studies can further deepen this research from three key aspects. First, combined with single-cell sequencing and *in vivo* imaging technologies, the differential molecular regulatory networks of hepatorenal injury were analyzed. The key mechanisms were clarified to provide a theoretical basis for precise targeted intervention. Meanwhile, it is imperative to break through the practical application bottlenecks of natural products in this field. Through structural modification and nano-delivery system, the targeting and bioavailability of active ingredients are improved. Based on the technology of multi-omics, the synergistic mechanism of “multi-component-multi-target-multi-channel” is expounded to solve the problem of vague action mechanism. Improve the research transformation system, establish the quality control standard of natural products and the evaluation model of hepatorenal damage. Carry out a large sample clinical controlled study to clarify its clinical application scenarios. Additionally, attention should be paid to the combined damage caused by compound toxicants and the barrier heterogeneity among special populations. Overall, greater efforts should be made to advance the integration of basic research and translational application, and further promote the standardized application of natural products.
